# Use of Aptamers as Diagnostics Tools and Antiviral Agents for Human Viruses

**DOI:** 10.3390/ph9040078

**Published:** 2016-12-16

**Authors:** Víctor M. González, M. Elena Martín, Gerónimo Fernández, Ana García-Sacristán

**Affiliations:** 1 Departamento de Bioquímica-Investigación, Instituto Ramón y Cajal de Investigación Sanitaria (IRYCIS)-Hospital Ramón y Cajal, 28034 Madrid, Spain; m.elena.martin@hrc.es; 2 Aptus Biotech SL, c/Faraday, 7, Parque Científico de Madrid, Campus de Cantoblanco, 28049 Madrid, Spain; g.fernandez@aptusbiotech.com (G.F.); garciasaa@aptusbiotech.com(A.G.-S.)

**Keywords:** aptamer, diagnosis, ebola, HBV, HCV, HIV, influenza, SELEX, therapeutic, virus

## Abstract

Appropriate diagnosis is the key factor for treatment of viral diseases. Time is the most important factor in rapidly developing and epidemiologically dangerous diseases, such as influenza, Ebola and SARS. Chronic viral diseases such as HIV-1 or HCV are asymptomatic or oligosymptomatic and the therapeutic success mainly depends on early detection of the infective agent. Over the last years, aptamer technology has been used in a wide range of diagnostic and therapeutic applications and, concretely, several strategies are currently being explored using aptamers against virus proteins. From a diagnostics point of view, aptamers are being designed as a bio-recognition element in diagnostic systems to detect viral proteins either in the blood (serum or plasma) or into infected cells. Another potential use of aptamers is for therapeutics of viral infections, interfering in the interaction between the virus and the host using aptamers targeting host-cell matrix receptors, or attacking the virus intracellularly, targeting proteins implicated in the viral replication cycle. In this paper, we review how aptamers working against viral proteins are discovered, with a focus on recent advances that improve the aptamers’ properties as a real tool for viral infection detection and treatment.

## 1. Introduction

Viruses are infectious agents that enter and replicate only inside the living cells of other organisms. Once the virus replicates inside the cell, it may remain dormant for long periods of time or be released immediately and attach to other healthy cells to begin the infection process again. Many diseases are caused by viruses such as the influenza, hepatitis, human immunodeficiency virus (HIV) or emerging viral diseases. While they differ in symptoms such as fever and weakness, some present no symptoms at all. Rapid and secure diagnosis of viral infections is a key factor for treatment of these diseases avoiding new spread.

Viruses cause different types of damage to the body which, if left untreated, can lead to death. There are many antiviral drugs that block the infection process at different stages. Some drugs prevent the virus from interacting with the healthy cell by blocking a receptor that helps internalize the virus into the cell. Other drugs inhibit the proliferation of the virus within the cell. The simultaneously use of several drugs affecting different processes increases the probability of recovery of the patient. Although some viral infections such as hepatitis or HIV remain latent for a long time current treatments control the virus and prevent further damage to the body.

Viral infections usually produce an immune response in the host that eliminates the infecting virus. The same protective effect is produced by vaccines, which confer an artificially acquired immunity to the viral infection. However, some viruses including those that cause acquired immune deficiency syndrome (AIDS) and viral hepatitis evade these immune responses and result in chronic infections. Antibiotics have no effect on viruses, but several antiviral drugs have been developed. Because viruses use vital metabolic pathways within host cells to replicate, they are difficult to eliminate without using drugs that cause toxic effects to host cells in general. The most effective medical approaches to viral diseases are vaccinations to provide immunity to infection, and antiviral drugs that selectively interfere with viral replication.

Most of the antiviral drugs are nucleoside analogues which lack the hydroxyl groups. Viruses mistakenly incorporate these analogues into their genomes during replication and, in consequence, the newly synthesized DNA is inactive and the life-cycle of the virus is then halted. Some of the most frequently prescribed antiviral nucleoside analogues based-drugs are aciclovir for Herpes simplex virus infections and lamivudine for HIV and Hepatitis B virus infections [1]. During the last years, the nucleoside analogue drug ribavirin combined with interferon has been used for hepatitis C treatment [2], although currently there is a more effective treatment that includes simeprevir available for patients with genotype 1 and genotype 4 [3]. The treatment of chronic carriers of the hepatitis B virus by means of a similar strategy using lamivudine has been developed [4]. Today, the first line treatment of choice includes one of three drugs: Peg-IFN, entecavir or tenofovir because of their greater power and because they produce a very low rate of resistance.

## 2. Aptamers: A Potential Diagnostic and Therapeutic Alternative

Novel anti-viral agents have emerged from the field of in vitro selection of nucleic acid aptamers [5,6]. Aptamers are single-stranded folded nucleic acids (RNA or ssDNA) able to specifically recognize a target molecule with high affinity. The term aptamer, derived from the Latin word “aptus” which means to fit, was firstly introduced by the Nobel laureate Szostak and Ellington [7] when they described the in vitro selection of RNA molecules that bind specifically to a variety of organic dyes. This in vitro process, called SELEX (Systematic Evolution of Ligands by EXponential enrichment), was developed by Gold and Tuerk [8].

**Figure 1:**
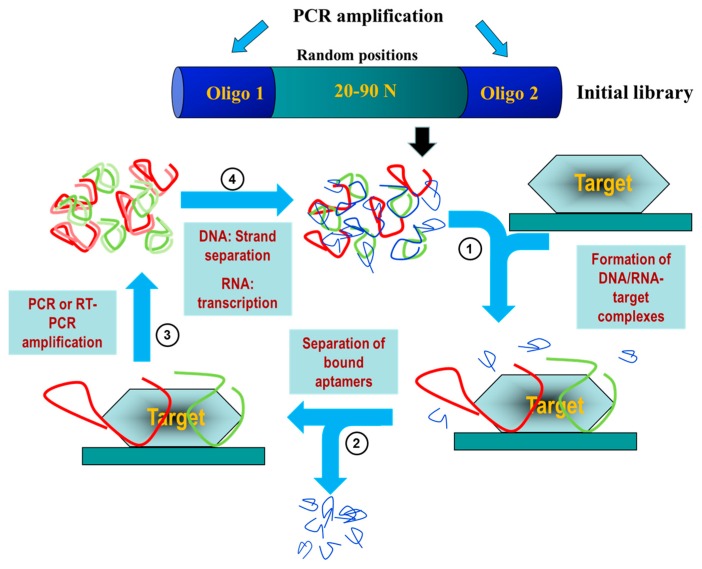
Scheme showing characteristics of each aptamer molecule in the population and the basic steps of a SELEX process. Each aptamer contains a central region with random sequence flanked by constant 5′ and 3′ ends that serve as primers. The SELEX method consists in iterative cycles of in vitro selection with several critical steps: (**1**) incubation with the target; (**2**) removal of unbound sequences; (**3**) amplification of bound sequences; (**4**) reconstitution of single stranded DNA or RNA. Nucleotides (N), Polymerase chain reaction (PCR); Reverse transcription polymerase chain reaction (RT-PCR).

The SELEX process begins with the synthesis of an oligonucleotide library consisting of a central region with random sequence flanked by constant 5′ and 3′ ends that serve as primers (Figure 1). Every member of the library is a linear oligonucleotide with a unique sequence that acquire three-dimensional structure depending on the experimental conditions (pH, ionic strength, temperature, etc.) or the presence of a ligand [9]. These highly structured aptamers are capable of binding to the target with high affinity and specificity. The diversity of an oligonucleotide library depends on the number of random nucleotides that contains each oligonucleotide molecule. Thus, an oligonucleotide library whose molecules contain a random sequence of 40 nucleotides (4^40^) would be represented by 1.2 × 10^24^ different sequences. However, in practical terms, the complexity of a combinatorial library of oligonucleotides is limited to 10^12^–10^18^ different individual sequences [10].

The first selection step implies the incubation of the target molecule with the initial library in the optimized conditions (temperature, pH, salt concentration) for the final use of the aptamer [9]. During the selection step, a subpopulation of individual sequences specifically interacts with the target molecule and this enriched subpopulation is isolated by physicochemical techniques [11,12,13]. Subsequently, bound sequences are eluted and amplified for a next round of selection in which the stringency of the selection conditions may be increased to identify the tightest-binding sequences. Iterative rounds are performed until the population is enriched with sequences that display high affinity and specificity for the target (Figure 1).

During the last 25 years, several modified SELEX processes have arisen that allow adapting the technology to different targets (whole cells, small molecules, organic dyes, peptides, etc.) or to different final applications. As a final point, the selected aptamers are produced by chemical synthesis and purified to a very high degree and, therefore, eliminating the batch-to-batch variation found when using antibodies. The main advantages of aptamers in relation to antibodies are summarized in Table 1.

RNA and DNA aptamers have specific characteristics that would give them advantages for different applications. For instance, the process of selection of DNA aptamers is shorter and the chemical synthesis of DNA oligomers is cheaper and easier than the RNA oligomers. In addition, DNA aptamers usually have greater chemical stability and longer shelf life than RNA oligomers and are more resistant to nucleases activity inside living organisms. These characteristics would seem to confer to DNA aptamers important advantages to be used. However, RNA aptamers seem to have greater flexibility when it comes to folding and acquiring tertiary structures and they can be genetically encoded, and so they can be expressed directly by the target cells or organisms. Although it is commonly accepted that RNA aptamers are preferably used for therapy, while DNA aptamers are preferentially used as diagnostic tools, there are some exceptions to this non-wrote rule [14].

Individual aptamers can be further modified in order to improve their approachability [12,15]. For instance, chemical modifications may be introduced rising the aptamer in vivo stability. Additionally, more variations may be needed for intended applications like biotin and/or digoxigenin labelling, fluorescent reporters, or any other chemical group (thiols, amines, etc.) required for biosensing or diagnostic purposes. Moreover, aptamers can be further truncated to eliminate the oligonucleotide sequences which are not important for the interaction with the target or for the correct three-dimensional aptamer structure [16,17]. Identification of truncated aptamers limited to the minimum domain of interaction requires considerable efforts even though aptamers of less than 40 nucleotides in length have been already reported [18,19,20]. 

## 3. Aptamers for Virus Diagnosis and Treatment

### 3.1. Aptamers against Human Immunodeficiency Virus (HIV)

Human immunodeficiency virus is a lentivirus (a subgroup of retrovirus) that causes HIV infection and over time AIDS [21]. HIV infects essential cells in the human immune system such as CD4^+^ T cells, macrophages, and dendritic cells. When CD4^+^ T cell numbers decline below a critical level, cell-mediated immunity is lost, and the body becomes progressively more susceptible to opportunistic infections. HIV enters cells by endocytosis through the interaction of gp120 viral surface protein (SU) with CD4 host cell. The subsequent interaction of this complex with chemokine coreceptors produces a conformational change in viral protein gp41 that promotes the fusion of virion and target cell membranes leading to the release of HIV particles into the cell. Once inside the cell, viral uncoating generates the viral reverse transcription complex, and the reverse transcription gives the HIV preintegration complex (PIC). The PIC gets into the nucleus and the HIV DNA (provirus) is integrated into the cellular chromosome. Integration can lead to either latent or transcriptionally active forms of infection. The latent form gives the viral latency in cells that can replicate in new infected cells; this provirus can remain hidden during years or replicate and form new viral particles in any moment. The transcriptional active forms are transcript and translated forming new viral particles that dead the host cell and goes to infect new cells. Great efforts are being made to get sensitive, fast and simple diagnostic methods and effective therapies.

#### 3.1.1. Aptamers HIV in Diagnostics

At present, the initial clinical testing for HIV in human is made with an antigen/antibody combination immunoassay that detects HIV-1 and HIV-2 antibodies and HIV-1 p24 antigen to screen for established infection with HIV-1 or HIV-2 and for acute HIV-1 infection [22]. Enzyme-linked immunosorbent assay (ELISA) and real time PCR are the methods used and accepted in clinical samples.

Aptamers that recognize HIV proteins, like Tat and Rev, have been mainly used in several biosensor systems based in Surface Plasmon Resonance (SPR) and quartz crystal microbalance (QCM) techniques. Thus, Tombelli et al. generated biosensors based on SPR and QCM technique. They introduced aptamers capable of detecting the HIV-1 Tat protein, using biotin-streptavidin interaction. This approach allows for high specificity distinction between Tat and Rev (other HIV protein, structurally similar to Tat) [23]. Other system target Tat protein using biosensor based on the diamond field-effect transistor (FET) technique [24]. These “apta-biosensors” have high sensibility and specificity but the devices are complex and expensive.

#### 3.1.2. Aptamers to HIV as Antiviral Agents

**Figure 2:**
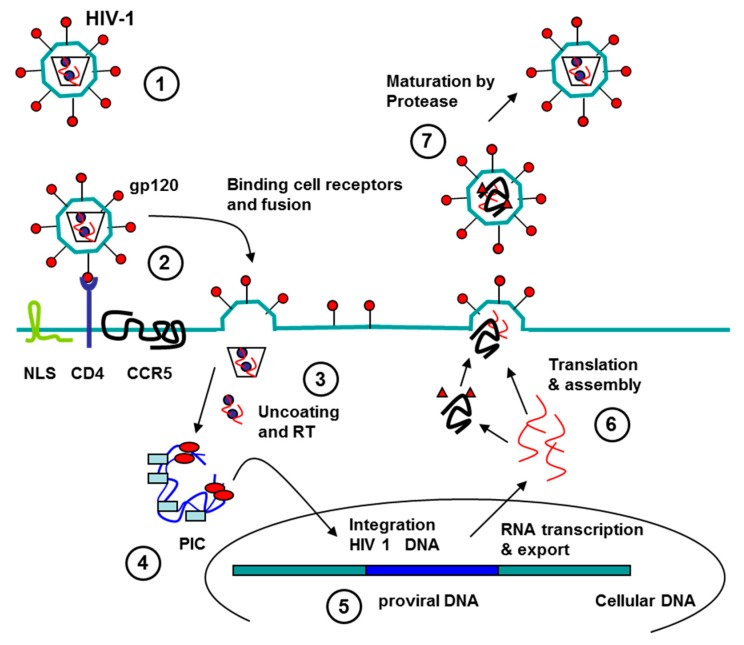
Scheme showing the targets for aptamers in HIV-1. (**1**) The HIV viral particle has an inner capside containing ssRNA viral genome and integrase and retro-transcriptase proteins, mainly; (**2**) The outer envelope has gp120 and gp40 proteins involved in interaction with cellular receptors (CD4, CCR5, NLS) and fusion to cellular membrane; (**3**) In the cytoplasm the ssRNA viral genome is released and the retro-transcription step is produced; (**4**) The integrase protein binds to dsDNA viral genome by LTR ends sequences and other cellular proteins forming the pre-integration complex (PIC); (**5**) The PIC goes into nucleus through the nuclear pore and is integrated in the cellular genome by the integrase protein activity (provirus); (**6**) The viral RNAs are transcripted from proviral DNA and exported to cytoplasm to translate viral proteins as protease and a big pre-protein that are assembled to new RNA viral genomes and leave the cell with outer envelope from the cellular membrane; (**7**) After the budding viral particle, the proteases process the big pre-protein and get a mature viral particle.

At present, the treatment HIV-1/AIDS is by a combination of several antiretroviral drugs (cART), which can slow the progress of the disease and reduce the risk of death and disease complication, but it is not curative. Moreover, many patients do not tolerate cART because it has severe side effects, and it is too expensive for patients in developing countries. In this regard, aptamers have been considered an alternative or adjuvant to the chemical antiviral agents in cART to overcome these limitations. To date, highly specific, nucleic acid-based aptamers that target various parts of HIV-1 genomes, HIV-1 proteins (including HIV-1 protease (PR), reverse transcriptase (RT), nucleocapsid, gp120, and Gag) and cellular proteins (nucleolin, CD4 or CCR5) have been isolated and shown to effectively suppress viral replication to apply in HIV therapy [5,25,26,27] (Figure 2).

##### **Aptamers** **to HIV Genome**

Long terminal repeats (LTRs) are sequences necessary for proper expression of viral genes. Interference with the function of these RNA domains either by disrupting their structures or by blocking their interaction with viral or cellular factors may seriously compromise HIV-1 viability. Srisawat and Engelke selected RNA aptamers that can bind to the LTRs of HIV-1 DNA [28]. They showed that conserved segments present in several of the aptamers could form duplexes via Watson-Crick base-pairing with preferred sequences in one strand of the DNA, assuming the aptamer invaded the duplex and, in consequence, these specific aptamers would inhibit the transcription process. Meanwhile, Sanchez-Luque et al. reported the in vitro selection of specific RNA aptamers against the 5′-untranslated region of HIV-1 genome. Those aptamers inhibited more than 75% of HIV-1 production in a human cell line. The analysis of the selected sequences and structures allowed for the identification of a highly conserved 16 nt-long stem-loop motif containing a common 8 nt-long apical loop (RNApt16; 5′-CCCCGGCAAGGAGGGG-3′) that produced an HIV-1 inhibition close to 85%, thus constituting the shortest RNA molecule so far described that efficiently interferes with HIV-1 replication [29]. The reason to use RNA aptamer is to go into the cell as DNA plasmid and to get intracellular expression of RNA aptamer to block the target.

##### **Aptamer** **to HIV Proteins**

###### Aptamers to Protease (PR)

During its life cycle, HIV must successfully complete several key steps in order to replicate in the host environment. One such critical step involves the processing of its Gag and Gag-Pol polyprotein precursors into their mature functional components during viral maturation. This function is provided by the virally encoded aspartyl protease (PR) and is thought to occur either during or immediately after budding [30,31]. In its active form, this protein is a 22 kDa homodimer consisting of two 99-amino acid long subunits each of which contributes a catalytic aspartate residue to the active site. Inhibition the proteolytic activity of PR leads to the production of immature and non-infectious particles. Duclair et al. have developed RNA aptamers against viral protease that inhibited HIV replication in vitro [32].

###### Aptamers to Integrase (IN)

Several small aptamers containing G-quadruplex selected against HIV proteins have demonstrated antiviral activity [33]. The viral enzyme integrase (IN) is essential for retroviral replication, catalyzing the integration of the newly synthesized double-stranded viral DNA genome into the host genomic [34]. Ojwang et al. obtained a modified aptamer, named T30177, able to inhibit integrase activity with IC50 values in the nanomolar range [35]. T30177 was the first IN inhibitor tested in clinical trials (Zintevir™, developed by Aronex Pharmaceuticals, The Woodlands, TX, USA) [36]. Derivatives of T30177, more stable than the parental molecule and also capable of efficiently inhibiting HIV-1 replication in cell culture have been later developed [37,38,39].

###### Aptamers to Reverse Transcriptase (RT)

Reverse transcriptase has two enzymatic activities, a DNA polymerase activity that can copy either a DNA or an RNA template, and an RNase H that cleaves RNA only if the RNA is part of an RNA/DNA duplex. The two enzymatic functions of RT, polymerase and RNase H, cooperate to convert the RNA into a double-stranded linear DNA [40]. DeStefano and Nair confirmed in vitro effectiveness of DNA aptamer, named 37NT, directed against the reverse transcriptase of HIV HXB2 strain. The aptamer competed with the natural template for the binding site in the enzyme, subsequently producing inhibition of the viral replication [41]. In parallel, Michalowski et al. identified three aptamers (RT5, RT6 and RT47) which contained a bimodular structure comprising a 5′-stem-loop module linked to a 3′-G-quadruplex. In addition, the authors demonstrated that this DNA aptamers inhibited RT from diverse primate lentiviruses with low nM IC_50_ values [42].

Another interesting approach is to inhibit the RNase H activity associated with RT. With this purpose, Andreola et al. identified two DNA aptamers with G-rich sequences, named ODNs 93 and 112, capable of forming G4 structures. These aptamers inhibited the RNase H activity of HIV-1 RT in vitro with IC_50_ values in the sub-micromolar range, while no effect was observed on cellular RNase H [43]. Shorter DNA aptamers derived from ODNs 93 and 112, named 93del and 112del, which maintained the capability to form stable G4 structures, were able to inhibit also HIV-1 integrase in the nanomolar range [44,45].

###### Aptamers to Nucleocapsid Protein

The nucleocapsid (NC) protein of HIV-1 plays an important role in the encapsidation of viral RNA and assembly of viral particle and the progress of some viral infections can be prevented by inhibition of nucleocapsid synthesis [46]. Since the NC protein is resistant for mutation, it might be an excellent target for the anti-viral therapy. Kim et al. isolated RNA aptamers that bind to the mature form of the NC protein with high affinity and compete for the packaging element (psi) RNA binding to the NC protein. Authors suggested that stabilized RNA aptamer is expected to act as an inhibitor for the viral packaging [47].

###### Aptamers to Surface Glycoprotein (gp 120)

Gp120 is essential for virus entry into cells as it plays a vital role in attachment to specific cell surface receptors mainly on helper T-cells. Several small aptamers containing G-quadruplex selected against gp120 have demonstrated antiviral activity [33]. The first of these molecules was the phosphorothioate 8-mer d(TTGGGGTT), named ISIS 5320, which forms a tetrameric G-quadruplex structure that binds the V3 loop of gp120 inhibiting virus entry [48]. Later on, Koizumi et al. synthesized a set of G-rich oligonucleotides and identified the hexadeosyribonucleotide d(TGGGAG), known as Hotoda’s sequence, [49,50]. Several authors have used the Hotoda’s sequence as a lead sequence to make a series of modifications at the 5′ and 3′ ends of the molecule or mutations in the sequence that allowed to find molecules with high anti-HIV activity [50,51,52,53,54,55,56]. 

Khati et al. in 2003 and Dey et al. two years later described the isolation of 2′-fluoropyrimidine-substituted RNA aptamers that bind specifically to the surface glycoprotein (gp 120) of HIV-1 that potently neutralized HIV-1 infectivity in human peripheral blood mononuclear cells [57,58]. One of these aptamers (B40) and its truncated form (B40t77) were further structural characterized [59]. Shortly later, these authors showed that the aptamer binds to the CCR5-binding site on gp120 in a relatively CD4-independent manner, providing a mechanistic explanation for its neutralizing potency [58,60].

###### Aptamers to Gag Protein

Other typical therapeutic target is Gag polyprotein, because of its low variability, as compared to other sequences of HIV-1 genome [61]. Two aptamers have been developed against this protein. Thus, Lochrie et al. isolated RNA ligands that bind to the HIV-1 gag protein in the regions corresponding to the matrix (MA) protein or to the nucleocapsid (NC) protein. Interestingly, although the sequence of the aptamer was different to the HIV-1 RNA packaging element (psi), it bound to NC region interfered with binding of psi [62]. Years later, Ramalingam et al. selected a new RNA aptamer against HIV-1 Gag protein, named DP6-12. This aptamer displayed 20-fold inhibition in the extracellular capsid levels and reduced cellular levels of mRNA for Gag, probably by perturbation of specific Gag-genomic RNA interactions [63]. These results suggest that RNA aptamers may provide a novel method for inhibiting HIV replication.

##### **Aptamers** **to Cell Proteins**

Another strategy to block HIV infection is to target human proteins in host cells, like the cell co-receptor that inhibits HIV entrance into the host cell, which may have less side effects.

###### Aptamers to CCR5

HIV-1 commonly uses C–C chemokine receptor type 5 (CCR5) or C–X–C chemokine receptor type 4 (CXCR-4) as co-receptors along with CD4 to enter target cells. Human CCR5 is an important co-receptor for macrophage-tropic virus expressed by T-cells and macrophages. Differences in CCR5 are associated with resistance or susceptibility to HIV-1. As an essential factor for viral entry, CCR5 has represented an attractive cellular target for the treatment of HIV-1. Thus, Zhou et al. have reported the selection of RNA aptamers against CCR5 using high throughput sequencing (HTS) to analyze the RNA pools from selection rounds 5 to 9. The individual sequences were classified into six major groups (Group 1–6). Group 2, 4 and 5 shared a conserved sequence, which is comprised of 10 nucleotides UUCGUCUG(U/G)G, named G3. The G3 activity was studied by a “prophylactic” HIV-1 experiment determining whether the aptamer would block HIV infectivity of R5 viruses in cell culture. The results showed that the G3 aptamer efficiently neutralized HIV-1 infectivity of R5 strains with IC_50_ about 170~350 nM [25].

###### Aptamers to Nucleolin (NCL)

Nucleolin (NCL) is a multifunctional cellular protein that is overexpressed in cancer cell membranes. NCL is involved in the very initial step of HIV-1 virion-cell recognition. NCL is in cellular fractions containing the HIV genome, viral matrix and reverse transcriptase in addition to in complexes with CD4 and CXCR4/CCR5 at the cell membrane. This supports the potential role of this protein in viral entry. In the absence of the cellular receptors as CD4 and CXCR4/CCR5, HIV-1 attachment can occur through coordinated interactions with heparan sulfate proteoglycans and cell-surface-expressed NCL.

AS1411 is a G-rich aptamer that form a stable G-quadruplex structure and displays antineoplastic properties both in vitro and in vivo [14]. The major molecular target of AS1411 is NCL. Perrone et al. tested whether the aptamer AS1411 was able to interfere with HIV-1 cellular entry using different HIV-1 strains, host cells and at various times post-infection [26]. The results demonstrated that AS1411 efficiently inhibited HIV-1 attachment/entry into the host cell in the absence of cytotoxicity at the tested doses.

##### **Use** **of Aptamers for Delivery of Therapeutic Molecules**

Aptamers can also serve as elements that selectively recognize and bind to defined cell types or tissues. By attaching drug molecules, the aptamers can be used to deliver cargo molecules to or into specific cells or tissues of interest [64]. In order to reach efficient RNAi activity, aptamer-siRNA conjugates must be successfully internalized and released into the cytoplasm where they can meet the RNAi machinery [65]. To improve the Dicer entry and processing of the siRNA, one pair of complementary guanosine and cytosine (GC)-rich “sticky bridge” sequences can be chemically appended to the 3′ end of the aptamer and one of the siRNA strands, respectively. Both the aptamer and siRNA portions are chemically synthesized and subsequently annealed via “sticky bridge” [66].

Through either covalent conjugation or physical assembly, different siRNA molecules have been successfully functionalized with aptamers against HIV-1 gp120 and the CD4 receptor to achieve targeted RNAi efficacy which relies on specific interactions between the aptamer and its receptor expressed on the targeted cells or tissue. Zhou et al. isolated RNA aptamers against the HIV-1(BaL) gp120 protein that were used to create a series of dual inhibitory function anti-gp120 aptamer-siRNA chimeras [67]. The authors also demonstrated that one of these anti-gp120 aptamer-siRNA chimera is specifically taken up by cells expressing HIV-1 gp120, and that the attached siRNA is processed by Dicer, resulting in specific inhibition of HIV-1 replication and infectivity in cultured CEM T-cells and primary blood mononuclear cells (PBMCs). Interestingly, both the aptamer and the siRNA portions in the aptamer-siRNA chimera have potent anti-HIV activities [68]. Finally, the authors tested the antiviral activities of these chimeric RNAs in a humanized mouse model with multilineage human hematopoiesis showing that treatment with either the anti-gp120 aptamer or the aptamer-siRNA chimera suppressed HIV-1 replication by several orders of magnitude and prevented the viral-induced helper CD4^+^ T cell decline [69]. In order to improve the utility of aptamers as siRNA delivery vehicles, the same authors chemically synthesized a modified gp120 aptamer that was complexed with three different siRNAs (HIV-1 tat/rev and two HIV-1 host cell proteins, CD4 and TNPO3), resulting in an effective delivery of siRNAs in vivo, knockdown of target mRNAs and potent inhibition of HIV-1 replication in a humanized mouse model [70]. Very interestingly, following ending of the aptamer-siRNA cocktail treatment, HIV levels rebounded facilitating a follow-up treatment with the aptamer cocktail of siRNAs that resulted in complete suppression of HIV-1 viral loads that extended several weeks beyond the final injection.

Another interesting approach to targeted delivery was developed by Zhu et al. [27]. In this work, the authors show that a DNA aptamer obtained from the conversion of a previously reported RNA aptamer could be used to deliver siRNA into CD4^+^ T cells specifically. This DNA aptamer was covalently conjugated to the sense strand of the siRNA targeting HIV-1 protease (HIV-PR) and the resulting DNA aptamer-siRNA chimera specifically entered into CD4^+^ T cells and efficiently knockdown the expression of exogenous HIV-PR gene. This study demonstrated that DNA aptamers with intrinsic stability had a greater potential to be used for siRNA delivery.

Table 2 shows information on the aptamers described against HIV.

A direct application of the use of aptamer-siRNA chimera has been proposed by Wheeler et al. [71]. They demonstrated that CD4 aptamer-siRNA chimeras (CD4-AsiCs) specifically block gene expression in CD4^+^ T cells and macrophages in vitro, in polarized cervicovaginal tissue explants, and in the female genital tract of humanized mice. Thus, CD4-AsiCs could be used as the active element of a microbicide to avoid HIV sexual transmission.

In summary, all these interesting results point out the use of aptamers for development of novel anti-HIV-1 therapies.

### 3.2. Aptamers against HBV

Hepatitis B virus (HBV) is a partially double-stranded DNA virus of the *Hepadnaviridae* family classified into eight genotypes from A to H. The main element of the viral particle of HBV virus and also the most characterized component is the hepatitis B surface antigen (HBsAg) [72].

#### 3.2.1. Aptamers HBV in Diagnostics

One of the current objectives in the diagnostic of HBV is to develop a daily screening assay with a short period of detection between infection and recognition. Therefore, Suh et al. have developed a fast and low cost detection test based on competitive binding assay combined with fluorescence resonance energy transfer (FRET) [73]. The assay was built with an aptamer selected against the hepatitis B virus surface antigen (HBsAg), the best characterize and most frequently used HBV marker [74]. The described aptasensor was approximately 40-fold more sensitive than the conventional method. In 2015, a new set of three different DNA aptamers was selected against HBsAg and applied to develop a chemiluminescence platform. The new aptasensor was designed with aptamers-conjugated to magnetic nanoparticles reaching a detection limit five-fold better than the current enzyme-linked immunosorbent assay (ELISA) kits used in hospitals [75].

#### 3.2.2. Aptamers to HBV as Antiviral Agents

During the last years, different strategies to inhibit HBV activity have been described using aptamers selected against diverse viral target proteins. First, in 2010, Liu et al. proposed as a therapeutic tool RNA aptamers selected against HBsAg protein that are able to discriminate between HbsAg-expressing liver cells and HBsAg-negative cells [74]. Later, Feng et al. described an innovative strategy to fight against HBV infection suppressing the ribonucleoprotein complex interaction between the reverse transcriptase (named P protein) and the RNA stem-loop (ε). With this purpose, they isolated individual RNA aptamers with high affinity for the recombinant truncated HBV P protein that compete with the ε RNA interaction, without cytotoxic effects in culture cells, suggesting a potential antiviral activity of described aptamers [76]. On the other hand, Zhang et al. have chosen HBV core protein (HBVc) as a target to isolate DNA aptamers to inhibit nucleocapsid formation. They have shown that selected aptamer Apt.No.28 prevents nucleocapsid assembly and viral replication in the hepatic cellular line HepG2.2.15 [77]. Finally, Orabi et al. have isolated DNA aptamers to recognize specifically the matrix binding domain (MBD) but not the domain mutated in the residue 126 (I126A). One of the isolated aptamers, AO-01, has been proposed as a therapy molecule by inhibition of the virion production interfering the interaction between the matrix domain and the MBD [78].

Table 3 shows information on the aptamers described against HBV.

### 3.3. Aptamers against HCV

HCV viral genome is a positive-strand RNA that contains approximately 9.6 kilobases and codified for a polyprotein of around 3000 amino acids. The HCV polyprotein maturation process mediated by proteases gives rise to 10 viral proteins, four structural (C, E1, E2 and p7) and six non-structural (NS2, NS3, NS4A, NS4B, NS5A and NS5B) [79]. To date, only three proteins (p7, NS4A and NS4B) have not been used as antiviral target for isolate specific aptamers.

#### 3.3.1. Aptamers HCV in Diagnostics

Aptamer-based biosensors are a promising diagnostic platform to allow HCV infection detection in early stages or in immunosuppressed patients. Thus, different groups have developed diverse aptasensors to improve diagnostic assay of HCV infection. First, Lee et al. developed a biosensor prototype that specifically recognizes the HCV core protein from sera of an infected patient using selected RNA aptamers against core antigen. The HCV viral particles were retained by the 2′-F aptamers immobilized in a 96-well plate and detected by sequential steps with anti-core and Cy3-labeled secondary antibodies [80]. Later on, Chen et al. developed an early diagnostic assay based on sandwich-ELISA to recognize HCV viral proteins using biotin-labelled DNA aptamers against HCV Envelope glycoprotein E2. The obtained results from infected patients showed a good correlation between viral genome quantification assay, HCV antibody detection and sandwich-ELISA aptasensor [81]. Afterwards, Shi et al. developed a similar platform for early detection, coating the bottom of the well with C7, a DNA aptamer against HCV core labelled with biotin, and HCV-core antibody conjugated with horseradish peroxidase (HRP) is applied over the surface. The platform was applied to the detection of the protein in sera from HCV-infected patients and showed a proportional relationship between amplified RNA copies and HCV core protein concentration [82]. Moreover, Wang et al. designed a rapid, easy-to-use diagnostic platform composed of lateral flow strips treated with thiol-DNA aptamers against HCV core antigen. HCV ELISA assay and core aptamer lateral flow strips showed positive coincidence rates when compared with HCV RNA amplification assay [83]. In an effort to develop a diagnostic test to monitor the infectivity of HCV samples, Park et al. have designed an ELISA-like assay replacing the capture and detection antibodies for DNA aptamers selected against HCV E2. The Enzyme Linked Apto-Sorbent Assay (ELASA) has been described to be used for qualitative and quantitative analysis of virus in infected samples [84]. Further, two laboratories have developed label-free aptasensor to eliminate the labelling step and simplify the HCV detection method. Hwang et al. have described a highly sensitive label-free aptasensor based on nanomechanical microcantilevers. The biosensors were able to measure the surface stress due to the interaction between immobilized RNA aptamers and the HCV helicase [85]. On the other hand, Roh et al. have developed a label-free diagnostic platform to detect and quantify the presence of HCV polymerase NS5B viral protein using conjugated streptavidin-biotin RNA aptamers on an Octet biosensor [86].

#### 3.3.2. Aptamers to HCV as Antiviral Agents

**Figure 3:**
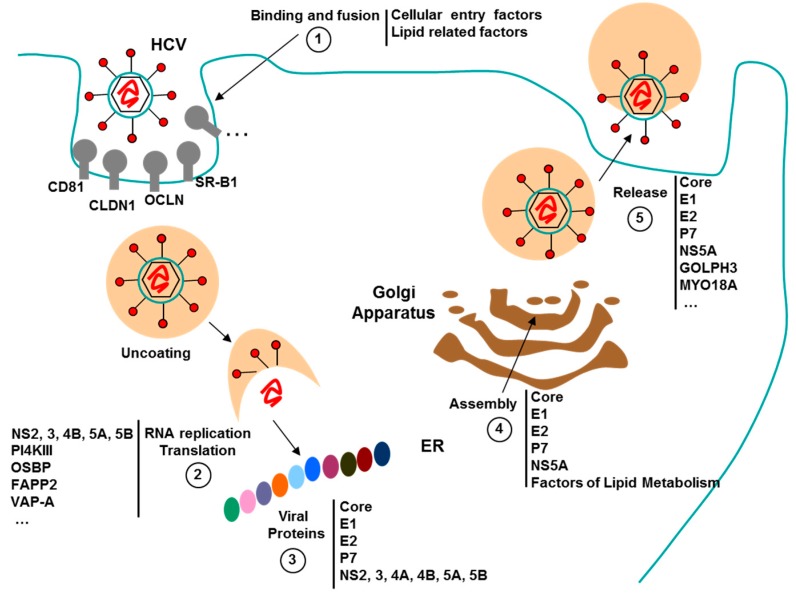
Scheme of Hepatitis C viral cycle showing the targets for aptamers. (**1**) Cellular entry factors and lipid related factors have been identified as important targets for aptamer selection, to avoid binding and viral fusion of HCV virus; (**2**) In the steps of RNA replication and translation several host factors as PI4KIII, OSBP or FAPP2 are candidates for aptamer selection; (**3**) HCV RNA genome encode for ten viral proteins, however until the date there are aptamers selected against seven of them; (**4**) Factors of lipid metabolism mediate viral assembly and can be chosen as targets for antiviral therapy; (**5**) Proteins and factors implicated in viral release are crucial for viral propagation and can be used for aptamer selection. Endoplasmic reticulum (ER).

Eradication of HCV disease is one of the main objectives of global public health. Currently, HCV infected patients are treated combining protease inhibitors, as Telaprevir (TVR) and Boceprevir (BOC), with pegylated-interferon and Ribavirin. However, the new direct-acting antivirals (DAA), TVR and BOC, generate a high rate of side effects, are too expensive and are also susceptible to new resistant viruses [87]. Therefore, it is necessary to develop new DAA treatments that are more effective and with fewer side effects than current therapies. To this end, different advances have been made based on aptamers against HCV and host cell proteins as therapy [88] (Figure 3).

##### **Aptamers** **to 5′ and 3′ Untranslated Regions (5′ and 3′UTR)**

Non-translated 5′ and 3′ regions have highly conserved sequences and structured regions closely related with transcription and replication of the HCV virus. Specifically, 5′ end contains the Internal Ribosome Entry Site (IRES) domain responsible of transcription initiation by ribosome recognition of HCV viral genome. However, 3′UTR includes a region essential for viral replication named *cis*-acting replication element (CRE). 

In 2003, Toulmé et al. decided to use subdomain IIId of IRES element as an antiviral target. They selected RNA aptamer and verified that isolated aptamers inhibit HCV translation in vitro and in cell culture [89]. In the same year, Kikuchi et al. isolated RNA aptamers capable of binding to the domain II of HCV IRES and showed that IRES-mediated in vitro translation was reduced from 20% to 40% by using the 2-02 aptamer [90]. Later, they isolated a new RNA aptamer population against the HCV IRES domains III-IV and corroborated that 3-07 aptamer had a high inhibitory effect on IRES-mediated translation in vitro and in vivo [91]. To improve the inhibitory effect of selected aptamers, they constructed two new molecules, named 0207 and 0702, composed by 2-02 and 3-07 aptamers linked by their ends. The fused aptamers recognized two different subdomains of IRES element and are at least 10 times more efficient than the parental aptamers in the inhibition of mRNA IRES-dependent translation in vitro [92]. Following with IRES as an anti-viral target, Romero-López et al. described an innovative in vitro selection method to isolate aptamers fused to a hammerhead ribozyme with capacity to inhibit RNA translation mediated by IRES. Selected chimeric aptamer-ribozymes were able to recognize the IRES element and cleavage the 5′ end at nucleotide position 363 [93]. The success of combining two functional elements in the same molecule was shown in the selected chimeric molecule HH363-50. Thus, the aptamer-ribozyme chimera did anchor to domain IV of the IRES element and inhibited in vitro and in vivo IRES-mediated translation [94]. Therefore, recruitment of ribosomal particles mediated by the IRES element was inhibited by the chimera HH363-24 that prevented both translation and replication in a hepatic cell line [95]. Moreover, to avoid HCV genome replication, Konno et al. isolated RNA aptamers against the 3′ end of the negative strand of the virus genome [96,97]. Interestingly, a RNA aptamer, named AP30, was able to recognize this minus-IRES region and reduce positive-strand genomic RNA synthesis [96]. To inhibit HCV replication, Marton et al. selected RNA aptamers against CRE element that were able to repress replication of HCV replicon in hepatic cells [98]. Subsequently, two selected aptamers, P58 and P78, interact with subdomain 5BSL3.2 of the CRE element and produce a structural reorganization of the 3′ end HCV genome and a significant decrease of HCV replication in vivo [99].

##### **Aptamers** **to HCV Proteins**

###### Aptamers to Nonstructural Protein 2 (NS2)

NS2 protein is a remarkable HCV target for the development of direct-acting antivirals due to the fact that NS2 protein is implicated in HCV replication and is essential in viral cycles. Recently, Gao et al. have isolated DNA aptamers against NS2. Specifically, they have shown that aptamer NS2-2 inhibited viral particle production as a result of N-terminal protein domain interaction, preventing NS5A/NS2 complex [100].

###### Aptamers to Nonstructural Protein 3 (NS3)

Multifunctional enzyme NS3 is an essential protein for virus survival and it is considered a good target for the development of new antiviral-drugs. The protease activity of the protein is found in the N-terminal domain and the helicase activity is present in the C-terminal domain of the enzyme. During the last years, the group of Nishikawa has been working with different sets of RNA aptamers against the full-length or truncated NS3 protein to inhibit its dual-activity. Initially, they isolated NS3-specific aptamers that inhibited the protease activity (10-G1) or the dual activity, protease/helicase, of the enzyme NS3 (G6-16 and G6-19) [101,102]. Afterward, new RNA aptamers against the helicase domain of NS3 protein, named G9-I, II and III, were able to inhibit protease activity in vitro [103]. Later on, the authors described the interaction between G9-I aptamer with Arg161/Arg130 residues in the truncated NS3 form as a putative target for protease activity inhibition [104]. To stabilize and protect G9 aptamers against exonuclease activity in vivo, Nishikawa et al. conjugated G9-II aptamer to the stem IV region of the Hepatitis delta virus (HDV) ribozyme. In addition, to allow nuclear export of the aptamer, the chimeric molecule HDV-G9-II (HA) was fused to a constitutive transport element (CTE) generating HAC molecule. Finally, the protease-inhibition capability of G9-II aptamer was checked, using HA and HAC expression vector in vivo [105,106]. In order to inhibit the dual activity of NS3, a poly U tail in the 5′ or 3′ ends of G9-I aptamer (5′-14U-NEO-III or NEO-III-14U) was added. The two constructions were able to inhibit with high efficiency of the protease and helicase activities of NS3. Moreover, NEO-III-14U decreased the interaction between NS3 protein and the 3′end of the positive or negative sense HCV RNA and inhibited protease activity of NS3 in vivo [107]. Next, a new set of RNA aptamers were selected against the helicase domain using the truncated NS3 protein (NS3h) and the aptamer with greatest capability to deplete helicase activity in vitro was identified and named aptamer #5 [108]. Finally, a dual-functional aptamer named G925-s50 was designed using a truncated version of aptamer #5 plus G9-II aptamer linked by 50 mer poly(U) spacer. The designed molecule G925-s50 showed a significant inhibition of NS3 helicase-protease activity in vivo and is proposed by Nishikawa group as the best candidate for anti-HCV therapy [109].

###### Aptamers to Nonstructural Protein 5A (NS5A)

It has been reported that NS5A protein is essential for HCV production and replication. Recently, Yu et al. have isolated and characterized DNA aptamers against HCV NS5A protein. Particularly, selected aptamer NS5A-5 was able to inhibit HCV virus infection by prevention of protein-protein interactions between NS5A and core protein [110].

###### Aptamers to Nonstructural Protein 5B (NS5B)

HCV nonstructural protein 5B (NS5B) is a RNA-dependent RNA polymerase protein (RdRp) responsible to the generation of positive-sense genomic HCV RNA and negative-sense RNA template. Reduction of HCV NS5B polymerase activity affects HCV viral life cycle and is one of the main objectives to isolate aptamers against NS5B. Thus, Biroccio et al. identified specific RNA aptamers against a truncated protein NS5B-Δ55 without the C-terminal region. One of the selected aptamers, B.2, blocked RNA transcription but not competed with the complex RdRp-RNA, using different binding site than RNA template to the NS5B protein [111]. In the same way, Bellecave et al. selected DNA aptamers against the NS5B viral protein. One of the chosen aptamers, 27v, competed with positive and negative sense HCV viral RNA to bind RdRp polymerase and blocked initiation and elongation steps of RNA transcription [112]. However, 127v aptamer partially competes to dissociate RdRp-RNA complex formation and only inhibited initiation steps of HCV transcription [113]. Moreover, interference of viral production and transcription inhibition of HCV virus was confirmed in vivo using 27v aptamer.

Table 4 shows information on the aptamers described against HCV.

Five years later, another set of RNA aptamers against NS5B protein were obtained by Lee et al. [114]. To avoid aptamer degradation, oligonucleotides were modified with 2′ hydroxyl (R–OH) or fluoropyrimidines (R–F). The R–OH aptamers blocked RNA synthesis of HCV replicon in cell culture without emergence of virus escape mutant or cellular toxicity. On the other hand, R–F oligonucleotides were truncated and conjugated with cholesterol- or galactose-PEG molecules to allow direct and specific liver delivery into cells or tissue. Cholesterol- and Gal-PEG-R–F t2 conjugated aptamer blocked RNA synthesis of HCV genome [115]. The above mentioned aptamers were non-genotype-specific; however, Jones et al. described for the first time aptamers against NS5 protein that exclusively recognized and inhibited RNA-polymerase activity of HCV virus subtype 3a [116].

###### Aptamers to Structural Proteins E1, E2 and Core

Envelope E1 and E2 glycoproteins are putative targets in therapy due to its role in HCV viral recognition to enter into hepatic cells. Chen et al. have isolated DNA aptamer against E2 glycoprotein. The selected aptamers have higher affinity to genotype 1a, 1b and 2a than others, and strongly prevented HCV viral infection in Huh7 5.1 cells [81]. Afterwards, Yang et al. described the potential antiviral action of DNA aptamers selected against E1E2 protein by HCV infection suppression in HuH7.5 cells without innate immune response action [117]. In the case of core, an essential protein for HCV viral assembly, Shi et al. have applied for therapy the above-mentioned aptamers in diagnostics. In Huh7.5 cells, the aptamers against HCV core protein repressed viral production as a result of defective assembly of virus particle without stimulation of innate immune response [82].

Finally, an innovative approach to inhibiting HCV viral infection has been designed based on sequestration of miR-122, implicated as a regulator of fatty-acid metabolism in mammals, exclusively in infected cells. To this end. Lee et al. have generated a chimeric molecule composed by a hammerhead ribozyme conjugated with an aptamer against NS5B protein. After aptamer recognition of HCV infection, ribozyme domain released a sequence complementary to miR-122 producing the inhibition of HCV replication in Huh-7 cells [118].

Selected aptamers against HCV have been shown to be good tools for inhibiting viral protein activity. However, none has been tested in clinical trials because it is necessary to overcome a number of limitations such as degradation, biodistribution, immune response or cellular internalization. Nevertheless, recent publications have described different strategies to overcome aptamer restrictions using cholesterol or ribozyme conjugation [115,118].

### 3.4. Aptamers against Human Papilloma Virus (HPV)

Human papillomavirus (HPV) is a DNA virus from the *papillomavirus* family. Most HPV infections cause no symptoms and resolve spontaneously, but some of them persist and result in warts or precancerous lesions which increase the risk of cancer of the cervix, vulva, vagina, penis, anus, mouth, or throat [119,120].

#### 3.4.1. Aptamers to HPV in Diagnostics

Several proteins from human papillomavirus, particularly E6 and E7, promote tumor growth and malignant transformation and are frequently associated with cervical cancer. Thus, these proteins represent ideal targets for diagnostic and therapeutic strategies. Belyaeva et al. reported two RNA aptamers to E6, named F2 and F4, which induced apoptosis in cells derived from an HPV16-transformed cervical carcinoma. This aptamers were able to inhibit the interaction between E6 and PDZ1 from Magi1, with F2 being the most effective inhibitor, while none of them inhibited E6–p53 interaction or p53 degradation [121].

Toscano-Garibay et al. isolated an aptamer (G5α3N.4) that exhibited specificity for E7 with a Kd comparable to aptamers directed to other small targets [122] that may be used for the detection of papillomavirus infection and cervical cancer. The same group characterized an RNA aptamer, named Sc5-c3, that recognized baculovirus-produced HPV-16 L1 virus-like particles (VLPs) with high specificity and affinity (Kd = 0.05 pM). This aptamer produced specific and stable binding to HPV-16 L1 VLPs even in biofluid protein mixes and thus it may provide a potential diagnostic tool for active HPV infection [123]. Recently, Graham and Zarbl identified several DNA aptamers that have high affinity and specificity to the non-tumorigenic, revertant of HPV-transformed cervical cancer cells, which can be used to identify new biomarkers that are related to carcinogenesis produced by HPV [124].

All these reports show the potential benefits of the E6/E7 aptamers as potential diagnostic agents in the future.

#### 3.4.2. Aptamers to HPV as Antiviral Agents

E7 has been shown to bind a number of cellular proteins, including the cell cycle control protein pRb. In an interesting study, Nicol et al. selected an RNA aptamer, termed A2, with high affinity to HPV-16 E7 [125]. Transfection of this aptamer into HPV-16-transformed cells resulted in induction of apoptosis and consequent inhibition of cell proliferation. In addition, the authors demonstrated that A2 bound to the N-terminal residues of E7 required for interaction with pRb and that, consequently, A2 disrupted the interaction between E7 and pRb in vitro [125]. Furthermore, the authors demonstrated that A2 enhanced E7 localization in the ER and that the A2-mediated reduction of E7 was not associated with proteasomal degradation suggesting that A2 perturbs normal E7 trafficking through promoting E7 ER retention [126].

For a different purpose, Gourronc et al. selected RNA aptamers that entered with significant internalization capacity (~5-fold) into HPV-16 E6/E7-human tonsillar epithelial cells (HTECs). In addition, although individual aptamers internalized into E6/E7 and primary HTECs with similar efficiency, one of them exhibited ~three-fold better internalization into E6/E7-HTECs. The authors claim that aptamers that internalize into cells may be useful for delivering therapeutic agents to HPV-16 associated malignancies [127].

### 3.5. Aptamers to Herpes Simplex Virus (HSV)

Herpes simplex virus 1 and 2 (HSV-1 and HSV-2) are two members of the herpesvirus family, Herpesviridae, that infect epithelial tissues before invading the nervous system, where it becomes latent. Unfortunately until now, it has not found any treatment to eradicate the virus [128].

Table 5 shows information on the aptamers described against HPV and HSV.

Aptamer technology has been used by Corbin-Lickfett et al. to identify RNA sequences capable of being recognized by HSV-1 ICP27 protein, an important regulator for viral gene expression. After SELEX procedure, GC-rich RNA sequences were isolated, which did not form stable secondary structures [129]. With a therapeutic purpose, Gopinath et al isolated two RNA aptamers (aptamer-1 and aptamer-5) against the ectodomain of the gD protein of HSV-1, which plays an important role in viral entry to the host cells. These aptamers specifically bind to gD protein of HSV-1 with high affinity but not the gD protein of HSV-2. Furthermore, aptamer-1 efficiently blocked the interaction between the gD protein and the HSV-1 target cell receptor (HVEM) in a dose-dependent manner with a EC_50_ in the nanomolar range. Anti-HSV-1 activity of aptamer-1 was analyzed by using plaque assays and the results showed that this aptamer efficiently inhibited viral entry. A shorter variant of aptamer-1 named mini-1 aptamer (44-mer) had at least as high an affinity, specificity, and ability to interfere with gD-HVEM interactions [130]. In a similar way, Moore et al. have reported the isolation and characterization of one aptamer, G7a, that binds the gD protein of HSV-2 and neutralizes infection through the Nectin1 and HVEM entry receptors with IC_50_ of 20 nM [131]. Interestingly, aptamers that prevent HSV-2 infection may also reduce the morbidity associated with HIV-1 as HSV-2 is a major risk factor for the acquisition of HIV-1.

### 3.6. Aptamers to Influenza

Influenza is considered the most prevalent infectious disease in humans. Three emerging influenza viruses were responsible for major pandemics in the twentieth century: the 1918 Spanish influenza virus, the 1957 Asian influenza virus, and the 1968 Hong Kong influenza virus [132]. Indeed, the 1918 Spanish influenza virus was estimated to have killed 20–50 million people worldwide [133]. More recently, a highly pathogenic avian virus of the H5N1 subtype has produced sporadic infections in humans and, while it is associated with high rates of mortality, its poor transmission in humans prevented a more extensive spread among human populations. However, in 2009, a new influenza A virus of the H1N1 subtype emerged (pH1N1) that possessed high transmissibility but relatively low virulence, rapidly spreading across the entire globe and causing the first pandemic of the 21st century [134,135]. Subsequently, 2013 witnessed the appearance of a new highly pathogenic avian virus of the H7N9 subtype in China [136].

Influenza viruses are enveloped RNA virus of the family *Orthomyxoviridae*. The virion surface carries two membrane glycoprotein components, hemagglutinin (HA) and neuraminidase (NA) and, in the central core, the viral RNA (negative-sense) genome fragmented into eight single-stranded molecules and viral proteins that package and protects this RNA. Each segment contains one or two genes that code for the 15 viral proteins. Highly variable surface proteins, HA and NA, are used to classify influenza subtypes. The combination of hemagglutinin and neuraminidase mainly determines the host organism and the viral infectiousness. Currently, 18 HA and 11 NA types have been identified being the subtypes H1, H2 and H3, and N1 and N2 commonly found in humans.

#### 3.6.1. Aptamers to Influenza Virus in Diagnostics

The detection rapid of influenza virus as well as the categorization of these viruses is particularly important due to the high risk of infection, the rapid propagation and the high frequency of mutation that often induces the arrival of new strains that can cause epidemics and even pandemics. An extensive review about the diagnostic strategies for influenza has been recently published [137].

The antibodies are the most common probe used to detect either the viral particles or host antibodies developed during the infection. However, although in most cases antibodies are able to distinguish between influenza A and B, only a few antibodies that differentiate subtypes of Influenza A or B have been reported. Alternative probes for subtyping are the aptamers. Thus, aptamers to hemagglutinin (HA) have been successfully and broadly used for the development on sensors for influenza detection. HA is expressed in high amounts in the viral surface and is required for binding and fusion with the host cell. Currently, more than 40 DNA and RNA aptamers to HA have been described since 2004, selected to recombinant hemagglutinins (H1, H3, H5, H9 and Ha from virus B) and to whole viruses (H5N1) (reviewed in [138]). Misono and Kumar selected an RNA aptamer against to HA of A/Panama/ 2007/1999 (H3N2) using SPR-based SELEX [139]. Gopinath et al. generated two RNA aptamers against intact influenza virus A/Panama/2007/1999 and HA of B/Johannesburg/05/1999. These RNA aptamers are able to discriminate among both A and B influenza viruses [140,141]. 

The recent advances in the development of rapid, automatic, point of care devices for the diagnosis and subtyping of influenza virus are sustained in two facts: (i) the rapid spread of influenza-associated H1N1 viruses that has caused serious concern in recent years; and (ii) H5N1 subtype of the avian influenza virus (AIV) caused the most lethal outbreaks of highly pathogenic avian influenza (HPAI) in poultry and fatal infections in human cases for over a decade. Thus, aptamers have been generated and found to be specific against these recent pandemic influenza viruses A/H1N1pdm [142] and H5N1 [143].

Lee et al. developed an integrated microfluidic system that was used to screen a specific aptamer for the influenza A/H1N1 virus in an automated and highly efficient manner [144]. The selected aptamer showed a specific and sensitive detection of the influenza A/H1N1 virus, even in biological samples such as throat swabs. Later, they used a new approach for fluorescence-based detection of the influenza A H1N1 virus using a sandwich-based aptamer assay that is automatically performed on an integrated microfluidic system [145]. The entire detection process was shortened to 30 min using this chip-based system which is much faster than the conventional viral culture method. The limit of detection was significantly improved due to the high affinity and high specificity of the H1N1-specific aptamers. In addition, this two-aptamer microfluidic system had about 10^3^ times higher sensitivity than the conventional serological diagnosis. The conformation of the aptamers changes in response to the solvent composition, including ion type and concentration, pH, and temperature. On the basis of this, Wang et al. have developed a microfluidic system that exploited the predictable change in conformation of the aptamer previously used in the group, exposed to different ion concentrations in order to detect multiple types of the influenza virus [146]. Thus, a single fluorescent-labelled aptamer is able to identify three different influenza viruses (influenza A H1N1, H3N2, and influenza B) at the same time, by modifying operating conditions, in 20 min. This chip-based aptamer-binding assay has several important advantages; it is rapid, accurate, and cheaper than multiple-aptamer screening.

Current methods for H5N1 AIV detection are virus isolation and RT-PCR that requires several days and expensive equipment and reagents. Rapid detection assays are also available (such as ELISA or immunochromatographic strips) but are less sensitive and specific. The alternative approach is biosensors technology, several biosensors have been developed to detect AIV among them biosensors using as probe aptamers (aptasensors) (reviewed in [147]. In the Li’s lab, a highly specific DNA aptamer that can bind H5N1 virus with high affinity was selected. Using this aptamer, other authors have developed different aptasensors based on Surface Plasmon Resonance (SPR) [148], a quartz crystal microbalance (QCM) aptasensor crosslinked polymer hydrogel [149] and several aptasensors based on impedance methods [150,151,152]. These aptasensors were able to detect H5N1 quickly and/or with more sensitivity than antibody-based biosensors. 

The impedance-based aptasensor described Fu et al. has the lowest detection limit, however, it requires signal amplification with labels and a prolonged detection limit [150]. The impedance aptasensor with microfluidics chips has a lower detection limit than the SPR-based aptasensor [148] and the same sensitivity as the QCM aptasensor [149], but the QCM-based aptasensors are not practical for in-field use due to the QCM’s predisposition to environmental noise. The major advantage of the impedance aptasensor with gold nanoparticles for signal amplification described by Karash et al. is that it requires a small sample volume and is cheaper than the detection platforms based on QCM or that use interdigitated electrode microfluidic chips [152]. Recently, Nguyen et al. developed a sandwich-type SPR-based biosensor for the detection of H5Nx viruses using a pair of aptamers selected against a mixture of H5Nx whole viruses using Multi-GO SELEX [153]. The sensitivity of the dual aptamer-based system increased by more than 50-fold than for single-aptamers. In addition, the sensitivity was additionally enhanced when the secondary aptamer was conjugated with gold nanoparticles.

#### 3.6.2. Aptamers to Influenza Virus as Antiviral Agents

**Figure 4:**
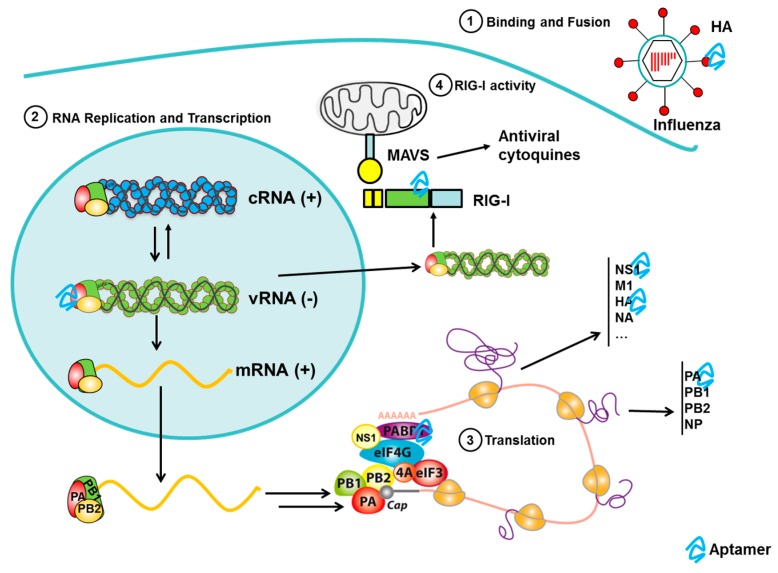
Scheme showing the protein targets for aptamers in influenza virus. (**1**) The viral particles, containing the segmented genome, present in its surface high amounts of hemagglutinin (HA) required for binding and fusion with the host cell; (**2**) The enveloped negative-strand viral RNA produces a full length (+) which in turn is copied to a full-length (−) strand RNA then used to assembled new virions. The vRNA serves as template for the synthesis of mRNA. The mRNAs of influenza virus possess a 5′ cap structure and a 3′ poly(A) tail that makes them structurally indistinguishable from cellular mRNAs; (**3**) Model of Influenza mRNA translation: The association of the viral polymerase, bound to the cap, and eIF4GI may be involved in the preferential translation of viral mRNAs during influenza infection. In addition, the interaction of NS1, bound to a conserved 5- untranslated region (UTR) element of the viral mRNA, with eIF4GI and PABP1 could promote the formation of a “closed loop” between the 5′ and 3′ ends of the viral mRNA; (**4**) RIG-I is a cytosolic receptor for non-self RNA that mediates immune responses against viral infections through IFNα/β production. Mitochondrial antiviral-signaling (MAVS) protein.

Several aptamers against influenza virus have been developed for therapeutics purposes, mainly targeting hemagglutinin (reviewed in [5]) (Figure 4). These aptamers are able to inhibit the entry of the virus of the cells by blocking hemagglutinin activity. The common technique to measure the inhibitory activity of the aptamers in vitro is the hemagglutination inhibition assay. The model was more extensively used to test the effect of the aptamers on the viral infection involving the use of cell cultures, mainly Madin-Darby canine kidney (MDCK) cells. The cells are infected with the virus and incubated with the aptamers and the inhibition of viral infectivity is tested. Using these assays, DNA and RNA aptamers selected against HA from Influenza A virus [142,154,155,156,157,158] or avian influenza virus [159,160,161], able to significantly decrease the viral infection in cells, have been described. However, only a few studies have described aptamers capable of mediating a reduction in viral pathogenicity in mice models. Jeon et al. evaluated the effect of the administration intranasal of the A22 aptamer, a DNA aptamer selected against the HA-(91–261) peptide, in mice before, at the same time and after virus infection [154]. The aptamer-induced inhibition of viral infection was determined by prevention of weight loss, decrease of viral load in the lungs and restriction of the level of inflammation and cellular infiltration. A22 reduced up to 95% of infection in all the strains tested (H1N1, H2N2 and H3N2). A22 was most effective when administered concomitantly with the viral infection leading to 95% reduction in viral burden. The administration of A22 one day prior to infection (preventive treatment) was less effective, probably because the DNA is partially degraded. Interestingly, the treatment with A22 two days following the infection (therapeutic treatment) still leads to almost 95% reduction in viral titer in the lungs of the mice. In 2014, Musafia et al. used A22 aptamer as a starting point and the quantitative structure–activity relationship (QSAR) tool to produce aptamers with 10–15 times more potent antiviral activity in animal models than A22 aptamer. The binding of these aptamers to the virus (20 times higher than A22) may not necessarily be sequence-specific being the most important properties the aptamer length, 2D-loops and repeating sequences of C nucleotides [157].

Other aptamers targeting NS1 or the PA polymerase subunit 1 have also been studied. NS1 is a nonstructural protein of small size, between amino acids 230 and 238 and with a molecular weight of 26 kDa. In view of its interaction with both RNAs and viral and cellular proteins, NS1 has been implicated in many of the alterations that occur during influenza virus infection. Moreover, NS1 has anti-interferon (IFN) properties leading to the inhibition of the host’s innate immunity [162]. Thus, the importance of NS1 in viral infection makes it an attractive therapeutic target. Woo et al. selected a DNA aptamer specific to NS1 that induced IFN-β production by inhibiting NS1 function. In addition, the selected aptamer was able to inhibit the viral replication without affecting cell viability [163].

Another antiviral strategy is the inhibition of the enzymes involved in the viral replication, transcription and translation. The polymerase complex of Influenza virus catalyzes the viral replication and transcription. This heterotrimer is composed of three subunits named PA, PB1 and PB2 [164,165,166]. PA plays the role of an endonuclease, cleaving host mRNAs downstream of their mRNA cap structures, which are recognized and bound by PB2 [167]. The N-terminal of the PA subunit (PA_N_), which holds the endonuclease activity site, is highly conserved among different subtypes of influenza virus, which suggests it is an attractive target in the development of anti-influenza agents. Yuan et al. selected DNA aptamers against both PA protein (three aptamers), and the PA_N_ domain (six aptamers) of an H5N1 virus strain [168]. Four of the six PA_N_ selected aptamers inhibited both endonuclease activity and H5N1 virus infection whereas the three PA-selected aptamers did not inhibit endonuclease activity and virus infection. Finally, one of the four effective aptamers, exhibited cross-protection against infections of H1N1, H5N1, H7N7, and H7N9 influenza viruses, with a 50% inhibitory concentration (IC_50_) around 10 nM.

Table 6 shows information on the aptamers described against influenza virus.

Vaccination is a powerful approach to diminish the effects of influenza epidemics, but the use of antiviral drugs can also be very useful, particularly in delaying the spread of new pandemic viruses. Neuraminidase inhibitors like oseltamivir, laninamivir, zanamivir, and peramivir are commonly used as antiviral agents to treat influenza infection, especially in Japan. However, because of the rapid increases in drug-resistant influenza virus, it is essential to develop new antiviral drugs as an emerging strategy to block cellular factors important for the infective cycle. The advantage of blocking important cellular pathways for the virus inhibitory effect is that, in principle, it is not specific of influenza strain and the emergence of resistant virus is minimized. A limited number of aptamers targeting host cell factors have been described. Of these, the use of RIG-I as a target for aptamers to control viral infection should be emphasized [169]. RIG-I is a cytosolic receptor for non-self RNA that mediates immune responses against viral infections through IFNα/β production [170]. The use of a specific RIG-I aptamer that activates RIG-I efficiently blocks the replication of the Newcastle disease virus, vesicular stomatitis virus and influenza virus in infected cells, evidencing that aptamers targeting cellular factors can act as efficient antiviral agents [169].

However, aptamers directed against cellular factors that establish essential interactions with influenza virus proteins had not been reported before. The mRNAs of influenza virus possess a 5′ cap structure and a 3′ poly (A) tail that makes them structurally indistinguishable from cellular mRNAs. However, selective translation of viral mRNAs occurs in infected cells through a discriminatory mechanism, whereby viral polymerase and NS1 interact with components of the translation initiation complex, such as the eIF4GI and PABP1 proteins [171,172,173]. Thus, the inhibition of viral protein–translation factor interactions or their destabilization can be potentially used as an antiviral strategy. Recently, Rodriguez et al. studied whether two aptamers which bind hPABP1 with high affinity (ApPABP7 and ApPABP11) are able to act as anti-influenza drugs [174]. Both aptamers inhibit influenza virus replication of H1N1 or H3N2 subtypes at high and low multiplicity of infection and the viral polymerase–eIF4GI interaction. In addition, aptamer ApPABP11 inhibits the interactions between NS1 and eIF4GI or PABP1. These results indicate that aptamers targeting the host factors that interact with viral proteins may potentially have a broad therapeutic spectrum, reducing the appearance of escape mutants and resistant subtypes.

### 3.7. Aptamers against Other Emerging Viruses

An emergent virus is a virus that has adapted and emerged as a new disease/pathogenic strain, with attributes facilitating pathogenicity in a field not normally associated with that of virus. This includes viruses that are the cause of a disease which has notably increased in incidence; this is often a result of a wide variety of causes from both the influence of man and nature. Most emergent viruses can be categorized as zoonotic (an animal disease that can be transmitted to humans), and this has the advantage of possibly having several natural reservoirs for the disease.

Most of these viruses have newly appeared in a population or have existed but are rapidly increasing in incidence or geographic range and only recently aptamers against emergent viruses such as Rift Valley Fever, Tick-borne encephalitis, Dengue, Ebola viruses or other arboviruses have been developed [175].

#### 3.7.1. Aptamers to Rift Valley Fever Virus (RVFV)

Rift Valley fever virus (RVFV) is a mosquito-borne bunyavirus (genus *Phlebovirus*) responsible for widespread outbreaks of severe disease such as hepatitis, encephalitis and hemorrhagic fever in humans [176]. The virus is endemic throughout much of the African continent. However, the emergence of RVFV in the Middle East, northern Egypt and the Comoros Archipelago has highlighted that the geographical range of RVFV may be increasing, and has led to the concern that an incursion into Europe may occur. At present, there is no licensed human vaccine [177].

The nucleocapsid protein (N) of RVFV is an RNA binding protein required for the production of viable virus because of its involvement in several stages of viral replication. This protein protects the viral genome from degradation and prevents the formation of double stranded RNA intermediates during replication and transcription by encapsidating viral genomic and antigenomic RNA [178]. Ellenbecker et al. isolated RNA aptamers that bound N with high affinity and identified GAUU and pyrimidine/guanine motifs in their sequences, which are also present within the coding region of the RVFV genome. Furthermore, the authors developed a truncated RNA aptamer labeled with fluorescein using a fluorescence polarization (FP) system. Titration of N with the 3′-FAM-labeled RNA aptamer gave an apparent Kd of 2.6 μM. Competitive binding experiments were conducted with four different aptamers and the apparent Ki values were all in the ~200 nM range. These data demonstrate that these aptamers might be used to construct a sensitive fluorescence based sensor of N binding with potential applications for drug screening and imaging methodologies [179].

#### 3.7.2. Aptamers to Tick-Borne Encephalitis Virus (TBEV)

Tick-borne encephalitis virus (TBEV) belongs to the family *Flaviviridae*, genus Flavivirus. This virus produces tick-borne encephalitis (TBE), an important emerging infectious disease that targets the central nervous system (CNS) [180]. There is currently no specific antiviral treatment for TBE because the specific immunoglobulin used in clinical practice has several disadvantages. The purpose of Kondratov et al. was to obtain an aptamer population against a fragment of the surface protein E of the TBEV, since it is available for aptamers outside of the host cell [181]. Authors showed that the treatment with the library of aptamers produced a TBEV neutralization index comparable with the results of neutralization of the commercial human immunoglobulin against tick-borne encephalitis (NPO Microgen, Russia). In addition, the enzyme immunoassay systems based on the immobilization of viral particles on antibodies are most commonly used for the TBEV diagnosis and the authors claim that protein E aptamers could substitute antibodies in these systems.

#### 3.7.3. Aptamers to Dengue Virus (DENV)

Dengue viruses (DENVs) belong to the *Flaviviridae* family, and contain four serologically and genetically distinct viruses, termed DENV-1, DENV-2, DENV-3 and DENV-4. The envelope (E) protein plays an important role in viral infection but, however, there is no effective antibody for clinical treatment due to antibody dependent enhancement of infection. Chen et al. identified an aptamer (S15) that can bind to DENV-2 envelop protein domain III (ED3) with a high binding affinity. S15 aptamer was found to form a parallel quadruplex structure that together with the sequence on 5′-end were necessary for the binding activity to a highly conserved loop between βA and βB strands of ED3. Although S15 aptamer was selected against DENV-2, the authors demonstrated that this aptamer can neutralize the infections by all four serotypes of DENVs [182].

The DENV capsid (C) protein functions as a structural component of the infectious virion but it may also have additional functions in the virus replicative cycle [183]. Balinsky et al. showed that the DENV C protein interacts and colocalizes with the multifunctional host protein nucleolin (NCL) and that this interaction can be disrupted by the addition of a NCL binding aptamer (AS1411), developed as AGRO100 by Aptamera (Louisville, KY, USA). Treatment of cells with AS1411 produced a significant reduction of viral titers after DENV infection. Moreover, the authors showed that treatment with AS1411 affected the migration characteristics of the viral capsid and identified a critical interaction between DENV C protein and NCL that represents a potential new target for the development of antiviral therapeutics [184].

From a technological point of view, aptamers have been used for efficient isolation of endogenously assembled viral RNA-protein complexes. Hence, Dong et al. developed an affinity purification strategy based on an RNA affinity tag that allows large-scale preparation of native viral RNA-binding proteins (RBPs) using the streptavidin-binding aptamer S1 sequence that was inserted into the 3′ end of dengue virus (DENV) 5′–3′ UTR RNA, and the DENV RNA UTR fused to the S1 RNA aptamer was expressed in living mammalian cells. This allowed endogenous viral ribonucleoprotein (RNP) assembly and isolation of RNPs from whole cell extract, through binding the S1 aptamer to streptavidin magnetic beads. This strategy led to identify several novel host DENV RBPs by liquid chromatography with tandem mass spectrometry (LC-MS/MS), including RPS8, which were further implicated in DENV replication [185].

#### 3.7.4. Aptamers to Ebola Virus (EV)

Ebola virus belong to the genus *Ebolavirus*. Four of five known viruses in this genus cause a severe and often fatal hemorrhagic fever in humans and other mammals, known as Ebola virus disease (EVD). Ebola virus has caused the majority of human deaths from EVD, and is the cause of the 2013–2015 Ebola virus epidemic in West Africa.

Viral protein 35 (VP35) is a multifunctional dsRNA binding protein that plays important roles in viral replication, innate immune evasion and pathogenesis. These multifunctional proteins offer opportunities to develop molecules that target distinct functional regions. With this purpose, Binning et al. used a combination of structural and functional data to determine regions of Ebola virus (EBOV) VP35 (eVP35) to target aptamer selection. Two distinct classes of aptamers were characterized based on their interaction properties to eVP35. These results revealed that the aptamers bind to distinct regions of eVP35 with high affinity (10–50 nM) and specificity. In addition, the authors showed that these aptamers compete with dsRNA for binding to eVP35 and disturb the eVP35-nucleoprotein (NP) interaction. Consistent with the ability to antagonize eVP35–NP interaction, select aptamers can inhibit the function of the EBOV polymerase complex reconstituted by expression of select viral proteins [186].

In many cases, aptamers have been used as a technological and research tool to identify RNA sequences that are recognized by different virus proteins. The zinc-finger antiviral protein (ZAP) is a host factor that specifically inhibits the replication of Moloney murine leukemia virus (MLV), Sindbis virus (SIN) and Ebola virus [187], by targeting the viral mRNAs for degradation and preventing the accumulation in the cytoplasm. With the aim to identify RNA sequences that could be a target of ZAP, Huang et al. used aptamer technology identifying G-rich RNA aptamers that contained conserved “GGGUGG” and “GAGGG” motifs in the loop region. Interestingly, overexpression of the aptamers significantly reduced ZAP’s antiviral activity and the substitution of the conserved motifs of the aptamers significantly impaired their ZAP-binding ability and ZAP-antagonizing activity, suggesting that the RNA sequence is important for specific interaction between ZAP and the target RNA [188].

#### 3.7.5. Aptamers to Severe Acute Respiratory Syndrome (SARS) 

Severe acute respiratory syndrome (SARS) is a disease caused by SARS coronavirus (SARS-CoV) that caused a pandemic pneumonia in 2002–2003, with a total of 8096 reported cases, including 774 deaths in 27 countries. SARS-CoV belongs to the Coronavirus genus in the Coronaviridae family and is an enveloped, positive-sense RNA virus with a genome of 27.9 kilobases. This RNA encodes two large polyproteins, pp1a and pp1ab, and four structural proteins including spike (S), membrane (M), envelope (E), and nucleocapsid (N) proteins. Pp1a and pp1ab are proteolytically cleaved into 16 non-structural proteins (nsps) that form the viral replicase–transcriptase complex (reviewed in [189]). 

Since the SARS outbreak, several diagnostic assays have been developed based on RT-PCR, ELISA or using biosensors. Nucleocapsid (N) protein is the target choice as it is one of the most abundant structural proteins. Both DNA and RNA aptamers against N protein have been developed with Kd of 4.93 and 1.65 nM, respectively [190,191]. DNA aptamer efficiently detects N protein by Western blot suggesting that it could be an alternative to the antibody. Ahn et al. have developed a nanoarray aptamer chip with the RNA aptamer selected as an antigen-capturing agent, which is able to detect N protein at a concentration as low as 2 pg/mL [190].

Only a few studies have been focused on the development of aptamers against SARS-CoV as antivirals, in spite of the fact that there is still no effective therapeutic treatment against the virus. Two studies have selected DNA and RNA aptamers against the non-structural nsp13 protein. This protein possesses NTPase, duplex RNA/DNA-unwinding and RNA-capping activities that are essential for viral replication and proliferation. These aptamers inhibited helicase activity with subnanomolar IC_50_, while the ATPase activity was not affected, suggesting that the aptamers may bind to the nucleic acid binding site of the helicase and block the unwinding activity [192,193].

Table 7 shows information on the aptamers described against emerging viruses.

## 4. Perspectives

Aptamer technology began to be developed in the early 90s and has seen major success in the last 10 years. However, despite all, the number of aptamers used in clinical practice is limited, probably due to the ignorance that still exists towards this technology and the advantage that the antibodies have in many diagnostic and therapeutic applications. Indeed, aptamers are molecules with extraordinary potential.

In the field of viral diseases, the number of drugs for treating these infections is very small and most of the available therapeutics are not very effective [194]. In addition, current diagnostic tools for viral infections are expensive and time consuming. These important diagnostic and therapeutic limitations have favored the development of aptamer-based systems, mainly because these show several interesting advantages in relation to antibodies. Thus, aptamers can recognize and/or inhibit target activity through specific and strong interactions superior to other biologics and small molecule therapeutics, with lower toxicity and immunogenicity profiles. In this sense, during the last years, aptamer technology is being used in a wide range of diagnostic and therapeutic applications associated with viral pathologies [195,196]. It is significant that aptamers selected as specific anti-viral molecules are effective in infected cells, however, none of the selected antiviral aptamers entered into clinical trials. In conclusion, it is necessary to continue research studies and successfully develop clinical trials to establish the use of aptamers as antivirals.

The success of treatment in viral diseases depends on the early detection of the infective agent. The most probable use of aptamers in virus diagnostics involves the development of more simple, fast and cheap diagnostics devices. One of these simple detection systems can be the Lateral Flow Immunoassays (LFIAs) which are currently used for qualitative monitoring in resource-limited or non-laboratory environments. The LFIA biosensing platform mainly comprises the sample pad and test pad, which is generally composed of nitrocellulose membrane, and provides a platform for both reaction and detection where the capturing molecules are antibodies [197]. Lateral Flow biosensing platform has been developed using an aptamer against hepatitis C virus (HCV) core antigen [83] and could be applied for HIV or emerging virus detection using aptamers against specific proteins. This method allows detection of viruses in endemic or transit of human areas. Another interesting approach to obtaining cheaper diagnostics/genotyping devices is using only one aptamer to detect several targets. From this point of view, the strategy by Wang et al. [146], in which they use the conformational change of one aptamer exposed to different ion concentrations to detect multiple types of the influenza virus could be used for genotyping of other viruses such as HBV or HCV.

From a therapeutic point of view, aptamers offer a hopeful solution in viral diseases because they can target elements of the virus or the infected host cell easier than the antibodies mainly due to their small size. The potential design of aptamers working against different targets might block the virion penetration into the cells or inhibit enzymes responsible for viral replication or other critical processes. In the case of HIV-1, despite efficient antiretroviral therapy, eradication of latent HIV-1 provirus is challenging and requires novel biological insights and therapeutic strategies. For this aim, novel target proteins should be chosen in HIV reservoir organs for the isolation of aptamers that could be applied to drug delivery or targeting of nanoparticles loaded with drugs to obtain HIV transcriptional activation.

As already mentioned above, RIG-I has been used as a target for aptamers to control viral infection [169]. Recently, Olagnier et al. investigated the inhibitory effect of a RIG-I agonist on the replication of Dengue and Chikungunya viruses [198]. The authors demonstrated that RIG-I stimulation generated a protective antiviral response against both pathogens. It would be motivating to study the use of RIG-I aptamers developed by Hwang for Dengue and Chikungunya therapy. Likewise, the effect of the aptamers developed by Guerra et al. against PABP in decreasing replication of influenza virus [174,199] could be studied in other viruses that also use the PABP of the infected cell [200].

Nanoparticles have been considered for a wide range of applications, both in soluble and insoluble forms. An advantage of soluble forms of nanoparticles is that they can encapsulate antibiotics/drugs and then release them when they reach the cellular environment, making them highly applicable for drug delivery systems. Studies on aptamer-based enhanced drug delivery have been reported for prostate cancer and lymphoblastic leukemia cells [201]. With this purpose, for HIV therapy, nanoparticles successfully loaded with the antiretroviral (ARV) drugs efficiently inhibited HIV-1 infection [202,203]. These results showed the benefit of the nanoparticles’ application to delivery of antiviral drugs to improve its bioavailability.

In conclusion, the use of aptamers in the development of diagnostic platforms or as therapeutic drugs is a promising alternative for the treatment of viral diseases.

## Figures and Tables

**Table 1:** Main advantages of aptamers over antibodies.

Characteristic	Advantage
Aptamers are identified through an in vitro process (SELEX)	Selection conditions can be manipulated to obtain aptamers stable in a wide range of environmental conditions
	Aptamers may be obtained against non-immunogenic proteins and toxins
Aptamers are produced by chemical synthesis	Little or no batch to batch variation
	Aptamers can be modified increasing their stability
	Reporter molecules can be attached to aptamers at precise locations not involved in binding
Aptamers are oligonucleotides	They can be amplified to be easily detected
	Denatured aptamers can be regenerated within minutes
	Aptamers are stable to long term storage and can be transported at ambient temperature
	They are not immunogenic
	Their small size allows for more efficient entry into the cell and its compartments

**Table 2:** Summary data on the aptamers selected against HIV discussed herein.

Virus	Name	DNA/RNA	Target	References
**HIV**	n.d.	DNA	Tat, Rev viral protein	[23,24]
	ASn, ALn, BSn, BLn	RNA	LTR viral sequence	[28]
	RNApt16	RNA	5′-untranslated region of HIV-1 genome	[29]
	PR10.1, PR10.9, PR10.13, PR10.18	RNA	Protease viral protein	[32]
	T30177, T30695	DNA	Integrase viral protein	[35,37,38,39]
	37NT	DNA	Reverse Transcriptase viral protein	[41]
	RT5, RT6 and RT47	DNA	Reverse Transcriptase viral protein	[42]
	ODNs 93 and 112 93del, 112del	DNA	RNAse activity associated to RT viral protein	[43,44,45]
	aptamers 8–6, 8–10, 8–13	RNA	Nucleocapsid viral protein	[47]
	Hotoda’s sequence and modifications	DNA	gp120 viral protein	[48,49,50,51,52,53,54,55,56]
	B4, B40, B40t77	RNA	gp120 viral protein	[55,56,57,58,59]
	ML6.8t33-82, ML8.20t14-75. DP6-12	RNA	Gag viral protein	[62,63]
	G3	RNA	CCR5 cellular receptor	[25]
	AS1411	DNA	NLC cellular protein	[14]
	n.d.	DNA	CD4 cellular receptor	[27]

n.d. = not determined.

**Table 3:** Summary data on the aptamers selected against HBV discussed herein.

Virus	Name	DNA/RNA	Target	References
**HBV**	anti-HBsAg RNA aptamer	RNA	HBsAg	[73]
	HBs-A22	RNA	HBsAg	[74]
	HO1, HO2, HO3	DNA	HBsAg	[75]
	Class I, An & Class II, Sn	RNA	MiniP protein	[76]
	Apt No 28	DNA	Core	[77]
	AO-01	DNA	Capsid	[78]

**Table 4:** Summary data on the aptamers selected against HCV discussed herein.

Virus	Name	DNA/RNA	Target	References
**HCV**	9-n	RNA	Core	[80]
	ZEn	DNA	E2 Glycoprotein	[81]
	C4, 7, 42, 97, 103 & 104	DNA	Core	[82]
	9-15	DNA	Core	[83]
	anti-HCVE2 aptamers	DNA (5-benzylaminocarbonyl-dUridine(Bz-dU)	E2	[84]
	RNA aptamer	RNA	Helicase	[85]
	biotinylated RNA oligonucleotide	RNA	NS5B	[86]
	2-02, 3-07, 0207 and 0702 aptamers	RNA	IRES element	[90,91,92]
	P6-n, HH363-n	RNA	IRES element	[93,94,95]
	Family I, II, III (AP30)	RNA	Minus-IRES domain I	[96,97]
	P58 and P78 aptamers	RNA	CRE element	[98,99]
	NS2-1, 2, & 3	DNA	NS2	[100]
	10G-1, G6-16, G6-19	RNA	NS3	[101,102]
	G9-I, G9-II, G9-III	RNA	NS3	[103,104]
	HDV-G9-II	RNA	NS3	[105,106]
	NEO-III, DNEO-III. NEO-III-14U, 5′-14U-NEO-III	RNA	NS3	[107]
	aptamer 5, 5 (1-30), 5ss, 5D, 5m, 5mS, 5mL1, 5mL2, ss5m-3′X, 3′(+) UTR, UC-3′X, 3′X	RNA	NS3	[108]
	NEO-35-sX, G925-sX	RNA	NS3	[109]
	NS5A-1,2,3,4 &5	DNA	NS5A	[110]
	A,1, A.2, B.1, B,2, B,3, C.1 & C.2	RNA	NS5B	[111]
	Class A, B, C & D (ODN n)	DNA	NS5B	[112,113]
	R-F, R-OH, Gal-PEG-R-F t2, Chol R-F t2, chol-aptamer	RNA	NS5B	[114,115]
	r10/N, r8a, r8b, r8c	DNA	NS5, genotipe 3a	[116]
	E1E2-1, 2, 3, 4, 5, 6	DNA	E1 E2	[117]

**Table 5:** Summary data on the aptamers selected against HPV and HSV discussed herein.

Virus	Name	DNA/RNA	Target	References
**HPV**	F2 and F4	RNA	E6	[121]
	G5α3N.4	RNA	E7	[122]
	Sc5-c3	RNA	HPV-16 L1 virus-like particles (VLPs)	[123]
		DNA	HPV-transformed cervical cancer cells	[124]
	A2	RNA	E7	[125,126]
		RNA	HPV-16 E6/E7-human tonsillar epithelial cells (HTECs)	[127]
**HSV**	GC-rich RNA aptamer	RNA	ICP27	[129]
	aptamer-1 and aptamer-5 mini-1 aptamer (44-mer)	RNA	gD protein	[130]
	G7a	RNA	gD protein	[131]

**Table 6:** Summary data on the aptamers selected against influenza virus discussed herein.

Virus	Name	DNA/RNA	Target	References
Influenza H3N2	Clone B	RNA	HA	[139]
Influenza H3N2	PN30-10-16 PN30-10-1	RNA	HA	[140,141]
Influenza H1N1	D26, D12	RNA	HA	[142]
Influenza H5N1	(1), (2), (3)	DNA	HA (4 cycles) and virus	[143,148,149,150,151,152]
Influenza H1N1	n.d.	DNA	virus	[144,145,146]
Influenza H5N1	n.d.	DNA	HA	[148]
Influenza H5Nx	IF10, IF15, IF20, IF22, IF23	DNA	Virus	[153]
Influenza H3N2	A21, A22	DNA	HA-(91–261) peptide	[154]
Influenza H5N1	A05, A10	DNA	HA from H5N1	[155]
Influenza H1N1	CP9P526, CP9P528, CP9P536, CP9P554, CP9P596	DNA	Sialic Acid Receptor (SAR) epitope	[156]
	BV42, BV35r	DNA	2nd generation (A22)	[157]
Influenza H1N1	1, 2	DNA	HA	[158]
Influenza H9N2	C7, C7-35M	DNA	H9 peptide (HA,101–257)	[159]
Influenza H5N1	8-1, 8-3, 8-10	RNA	HA	[160]
Influenza H9N2	A9, B4	DNA	HA	[161]
Influenza A	n.d.	DNA	NS1	[163]
Influenza H5N1	PAN-1, PAN-2, PAN-3, PAN-4, PAN-5, PAN-6	DNA	PA	[168]

n.d. = not determined.

**Table 7:** Summary data on the aptamers selected against emerging viruses discussed herein.

Virus	Name	DNA/RNA	Target	References
Rift Valley fever	n.d.	RNA	nucleocapsid protein	[179]
Tick-borne encephalitis	Population	DNA	surface protein E	[181]
Dengue	S15	DNA	DENV-2 envelop protein, domain III	[182]
Ebola	1G8–14, 2F11-14	RNA	protein 35 (VP35)	[186]
Ebola	21E, 21K, 21I	RNA	zinc-finger antiviral protein	[188]
Severe acute respiratory syndrome	n.d.	DNA/RNA	nucleocapsid protein	[190,191]
Severe acute respiratory syndrome	n.d.	DNA/RNA	non-structural nsp13 protein	[192,193]

n.d. = not determined.
